# New 3-dimensional implant application as an alternative to allograft in limb salvage surgery: a technical note on 10 cases

**DOI:** 10.1080/17453674.2020.1788698

**Published:** 2020-07-03

**Authors:** Jong Woong Park, Hyun Guy Kang

**Affiliations:** Orthopaedic Oncology Clinic, National Cancer Center, Korea

*Sir,—*I would like to congratulate Park et al. (2020a) for this excellent pilot study combining customised 3DiPC with conventional orthopedic prostheses for limb salvage surgery. Its a change in paradigm opening possibilities to adaptation, efficiency and cost minimisation. The concept is adaptable to situations and societies that struggle with allograft banking due to cultural, societal or financial overhead hurdles. Cost reduction will particularly resonate with institutes in low and middle income countries increasing the armamentarium. The method is especially attractive when achieving stable, durable fixation of small peri-articular fragments in children, adolescents and adults (Agarwal et al. 2010). Longevity of allografts, extra corporeal radiated bone and cryotherapy treated bone has unsolved challenges (Gerrand et al. 2003, Puri et al. 2018). They are prone to acute and delayed failure and do not allow immediate loading. The potential for bone ingrowth within the porous implant and further reduction in risk of aseptic loosening makes it biologically viable. While Park et al. have enumerated the key technical drawbacks and manufacturing challenges, a few technical details in combining them with available off the shelf prosthesis remain unanswered. For instance, the size, length and curvature of the intramedullary stem that would fit the residual native bone is difficult to predict. Would that enforce manufacturing of a wider bore 3DiPC, to combine with a conventional hip or knee implant, as a coupling agent, thus precluding a press fit/uncemented fit of the stem? The key factor, promising longevity, is the integrity of the bone implant interface, and cementation endagers that integrity. The use of lattice structure as a scaffolding in a metastatic case with the hope of bone ingrowth was curious as native biology is certain to lose against the incumbent cancer (Huang et al. 2017, Jenkins et al. 2017). Woud not an intercalary 3DiPC be significantly more robust and withstand post operative irradiation better? How does this compare with Trabecular metal, with outstanding results from the Mayo group in prior irradiated metastatic cases (Jenkins et al. 2017)? Was the size and structure of the lattice a constraint of design or the manufacturing process? Were attempts made to simulate the lattice structure of trabecular metal (Chen et al. 2016)? What concerns do they share about accelerated wear of titanium articulating with conventional chrome cobalt prosthesis, as in their pelvic reconstructions (Moharrami et al. 2013)? I look forward to a longer outcome analysis of this exciting new concept.

**Prakash Nayak**

Assistant Professor, Bone and soft tissue Unit

Department of Surgical Oncology

Tata Memorial Hospital

Homi Bhabha National Institute, Mumbai, India

*E-mail:* nayakprakash@gmail.com

*Sir,—*Thank you for the opportunity to respond to the letter from Professor Prakash Nayak, regarding our work entitled “New 3-dimensional implant application as an alternative to allograft in limb salvage surgery: a technical note on 10 cases.” Here, we present a more detailed description on the porous structure (3-dimensional [3D]-printed lattice), design process of the 3D-printed implant and prosthesis composite (3DiPC), and conjugation between 3D-printed and conventional orthopedic implants.

The effectiveness and safety of orthopedic implant fabricated using the 3D-printing technology remains to be proven and requires further accumulation of clinical experiences, but 3D-printed implant is promising especially in orthopedic oncology. Moreover, porosity with lattice structure can be easily implemented using the 3D-printing technology. The optimal pore size is defined as hundreds of micrometers before 3D printing (Bobyn et al. 1980, Mumith et al. 2017, Park et al. 2020b). For example, the trabecular metal (Zimmer biomet, Warsaw, Indiana, United States) has an open pore with the mean size of 440 um. The electron beam melting (EBM)-type 3D printer (ARCAM A1, Arcam AB, Mölndal, Sweden) used to fabricate implants reported in our paper has an accuracy of 200 µm. The pore size varied depending on the surgical location and load for endurance, and the lattice structure with 750-µm pore was often selected, considering the printing accuracy and metal powder removal after printing. Notably, the lattice unit size is different from the pore size. For example, as regards the dode-thin mesh (Magics 22, Materialise; Leuven, Belgium) we used, the actual pore size is 750 µm if the lattice unit size is 2 mm (Figure).

Bone incorporation is the key of implant longevity, and biocompatibility with the porous structure made of 64 titanium (Ti-6Al-4V) is well known from previous studies (Wu et al. 2013, Guyer et al. 2016, Mumith et al. 2017, McGilvray et al. 2018). For all patients we reported, the junction of the 3D implant to the host bone was designed to achieve a lattice structure depth of > 5 mm. To prevent interference of bone incorporation, PMMA bone cement was carefully applied to prevent it from entering the gap between the host bone and the implant. For some patients undergoing pelvic reconstruction involving the hip joint (patients 2, 3, 5, 6, 7), bone cement was used to attach the THA cup to the 3D-printed pelvic implant and simultaneously prepared outside of the surgical field while performing the surgical approaching procedure (Figure 1D in original paper). For patient 8, injectable bone cement was used to fill the gap between the 3D implant and the intramedullary nail after the 3D implant reduction to the host bone (Figure 1F in original paper). Trabecular metal (Zimmer biomet, Warsaw, Indiana, United States) is a porous tantalum implant with excellent surgical results and relatively long clinical experience. However, fabricating a patient-specific customized implant is more difficult with the trabecular metal than with 3D-printing lattice structure in orthopedic oncology. Remarkably, in revision hip surgery with a massive bone defect using trabecular metal, buttress, shim and restrictor are utilized (Zimmer biomet, Warsaw, Indiana, United States); bone cement is applied between the trabecular metal and revision shell of the hip joint, similarly with the 3DiPC method. Therefore, we believe that using a bone cement mantle with certain thickness is a more proven and realistic method than a press/uncemented fit to fasten 2 types of metal implants.

**Figure:**
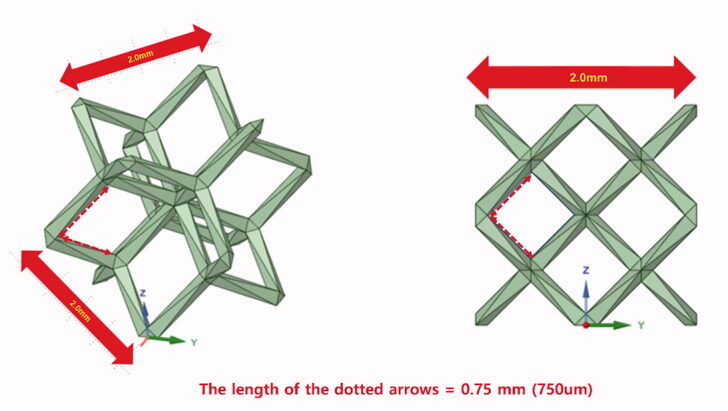
The unit and pore sizes in dode-thin lattice structure.

For metastatic bone tumor cases (patients 4 and 9), the bone biology near the tumor could be impaired by tumor itself or surgical procedures to achieve extended margin after curettage. Therefore, we selected candidates for the 3D implant limb salvage surgery with bone metastasis when a wide margin could be justified. Patient 4 already had a NED status preoperatively, and patient 7 had solitary metastasis in the femur diaphysis and underwent limb salvage surgery with wide margins. Therefore, the 3D implant was fixed to the healthy host bone in this case series.

In designing the 3D-printed implant for 3DiPC, special considerations should be made as to where the conventional implant will be combined. One of the disadvantages of the 3D-printed implant surgery is that the surgical plan including the bone margin cannot be adjusted. Therefore, the 3D-printed implant surgery should be carefully indicated for patients with rapidly growing tumors. Under the assumption that the surgery plan remains unchanged, a conventional implant used with 3D-printed implant should be confirmed during the designing process. For example, the diameter of the hip joint socket in a 3D-printed pelvic implant should be larger than that of the THA cup to achieve a 2-mm-thickness cement mantle. For 3DiPC with an intramedullary nail, the nail with the lowest curvature was selected, and the nail length and diameter were determined during the designing process preoperatively. Considering the nail insertion process, approaching direction, and nail curvature, the inner diameter of the 3D implant was increased as necessary so that the planned nail could be easily inserted and the gap between the 3D implant and the nail could be filled with bone cement. Nevertheless, nail insertion through the 3D implant is a difficult process. In patient 9, the mechanical strength of the implant itself was designed to be weak and wrapped with two pieces of the 3D implant around the nail.

A 3D-printed implant in 3DiPC technique is expected to have 2 main advantages over the structural allograft. First, no osteolysis-related problems are observed in a 3D-printed titanium alloy implant with a relatively constant mechanical property over time. However, aside from the absence of osteolysis, further mechanical study on the fatigue profile of the 3D-printed titanium alloy is needed. Second, surgical time can be reduced by omitting allograft trimming and simplification of fixation to the host bone. Pelvic bone tumor surgery with pelvic ring or hip joint reconstruction is large, time-consuming surgery associated with postoperative complications (Mankin et al. 2004). The surgical time cannot be directly compared because pelvic bone tumor surgery encompasses a wide range of surgeries depending on the specific surgical location, extent, and inclusion of the hip joint. In the original paper, the mean surgical time of pelvic reconstruction with hip joint (patient 1–7) was 160 (30–220) min, which is a relatively short time for pelvic reconstruction surgery.

We acknowledge that several aspects of 3D-printed implant surgery remain to be addressed in further studies with long-term follow-up. Currently, we believe that a 3DiPC method for joint reconstruction or strength security using conventional arthroplasty, tumor prosthesis, or intramedullary nail with well proven, stable performance is also a feasible option.

Jong Woong Park and Hyun Guy Kang *Orthopaedic Oncology Clinic, National Cancer Center, Korea* *E-mail:*jwpark82@ncc.re.kr*and*ostumor@ncc.re.kr
